# Editorial: How can diet impair thyroid function?

**DOI:** 10.3389/fendo.2022.1006547

**Published:** 2022-09-29

**Authors:** Caterina Mian, Carlo Foresta, Francesco Vermiglio

**Affiliations:** ^1^ Operative Unit of Endocrinology, Department of Medicine-DIMED; University of Padova, Padova, Italy; ^2^ Unit of Andrology and Reproductive Medicine & Centre for Male Gamete Cryopreservation, Department of Medicine-DIMED, University of Padova, Padova, Italy; ^3^ Division of Endocrinology, Department of Clinical and Experimental Medicine, University of Messina, Messina, Italy

**Keywords:** pfas, iodine, selenium, micronutrients, thyroid

It is well established that several natural compounds, micronutrients, vitamins, trace elements and new synthetic chemical compounds entering the food chain through industrial human activities (“endocrine disruptors”) can influence thyroid function. Recently published research on this topic was aimed at providing an updated view on how these compounds could have an impact on thyroid functional parameters.

An interesting contribution concerning diet, micronutrients and diet supplements came from the Gallo et al data. In their randomized controlled clinical trial (EudraCT 2017-00505011), the Authors investigated whether Selenium (Se) and cholecalciferol (VitD)) in addition to methimazole (MMI) might have been associated with hyperthyroidism, Graves’ disease (GD), owning to their antioxidant and anti-inflammatory properties (1, 2). Forty-two consecutive patients with newly-onset GD and marginal/insufficient Se and VitD levels were randomly assigned to treatment with either MMI monotherapy or MMI combined with Se and VitD. The Se treatment was withdrawn after 180 days, while the other treatments were continued. Combination therapies resulted in a significantly greater reduction in serum FT4 concentration and in better quality of life scores compared to the MMI group. The Authors concluded that reaching optimal Se and VitD levels normalizes thyroid function parameters in GD faster. Trans Fatty acids (TFAs) are unsaturated fatty acids containing one or more double bonds in the trans configuration. People are exposed to TFAs primarily through dietary intake of industrially-processed high-fat foods as well as animal products (3).

There has been controversial evidence regarding the effect of TFAs on thyroid function in animal studies, and epidemiological studies are lacking. Wang et al. investigated the potential associations between circulating TFAs and thyroid function biomarkers in a U.S. adult population. The Authors carried out a cross-sectional survey with 626 adults aged ≥20 years who participated in the National Health and Nutrition Examination Survey (NHANES) 2009–2010. The Authors’ findings revealed that TFAs exposure was correlated with thyroid function serum biomarkers, although more research is needed to evaluate the long-term health outcomes of these findings. A great deal of Literature shows that Omega-3 polyunsaturated fatty acids (PUFA) consumption appears to be beneficial on a number of clinical disorders, including autoimmune diseases. One major dietary source of PUFA is fish, particularly the small oily type of fish.

Unfortunately, fish (especially the large, top-predator fish like swordfish) are also a source of pollutants, including heavy metals, such as mercury. The review by Benvenga et al., recalled the effects on the thyroid through eating fish, also taking into account heavy metal interaction, and the interactions between the omega-3 PUFA and thyroid hormones on several levels. Whilst also pointing out that other natural compounds have shown benefits in the setting of thyroid autoimmunity, the review also looks at the commercial side of the benefits of other such substances, namely the expanding market of nutraceuticals. Overall, the Authors finally come to the conclusion that the use of supplements containing omega-3 in the clinical thyroid setting has enough scientific rationale. The incidence rate of thyroid cancer (TC) has gradually increased worldwide over the past several decades, which is potentially caused by individuals being excessively exposed to disease risk factors (4).

In recent years, multiple studies have revealed the underlying associations between intestinal flora and metabolites in thyroid cancer (5), although the synergistic mediation effects of the gut microbiota and its corresponding metabolites https://doi.org/10.3389/fendo.2022.893164 on lipid metabolism disorders in individuals with TC were not thoroughly screened. The aim of the Lu et al. study was to investigate the essential fecal species and metabolites and validate the coexistence relationship between intestinal microbiota and metabolites in patients with TC, through 16S rRNA gene sequencing and an integrated LC–MS-based metabolomics approach. The Authors observed that the diversity and richness of the gut microbiota in the TC patients were markedly decreased, with an altered composition of the gut microbiota. The results of this study are intriguing and may help to discover risk factors affecting the occurrence and development of TC in intestinal microecology.

Exposure to Per- and poly-fluorinated alkyl substances (PFAS), an environment-persistent emerging endocrine which disrupts chemicals, has been associated with the imbalance of thyroid hormones. However, little is known about the underlying cell mechanism causing this thyroid disrupting activity (6). Among PFAS chemicals, Perfluoro- octanoic acid (PFOA), perfluoro-octane sulfonic acid (PFOS) and the new generation substitutes, such as C6O4, are the most commonly employed. PFAS are used in a wide variety of consumer products and industrial applications because of their unique chemical and physical properties, so they are widely present in our daily life (7). De Toni et al. shed more light on this issue, studying the potential disrupting effect of PFOA, PFOS, and C6O4 on a murine thyroid cell model (FRTL-5), from the functional impact of PFAS exposure on cell function, involvement of cell toxicity, and analysis of membrane biophysical properties to the computational modeling of the possible interaction of PFAS with TSH-R along with subsequent experimental validation. The Authors observed that PFAS can differentially influence TSH dependent signaling pathways through the direct interaction with TSH-R. These results are preliminary and further studies carried out on human thyroid cell lines and animal models are required to confirm these interesting findings.

Another interesting study published in our Research Topic explored the effects of the Dutch Famine (1944- 1945) in late, mid, or early gestation on thyroid function parameters, in adulthood. Indeed, many data suggests the role of a foetal “environment” in the development of HPT axis setpoints: early-life exposures during gestation may permanently alter thyroid physiology and health in adulthood (8). Keestra et al., included 910 men and women registered as singletons at the Wilhelmina Gasthuis in Amsterdam in their study which comes from the Dutch Famine Birth Cohort(DFBC) a prospective birth cohort looking into the health of individuals born shortly before, during or after the Dutch Famine. Medical histories for previous diagnosis or current treatment for thyroid dysfunction were also investigated. The Authors found no differences in adult thyroid diseases at age 50 years due to parental famine exposure. However, the lower TSH levels in women exposed to famine in the second trimester of pregnancy suggests that there may be sex-specific effects from famine exposure during a critical period of thyroid development on hypothalmic-pituitary-thyroid axis regulation in adulthood.

In conclusion, articles in the present Research Topic provided more data which helped to get a better picture of several aspects of thyroid function interference which come from the diet and environment.

## Author contributions

All authors listed have made a substantial, direct, and intellectual contribution to the work and approved it for publication.

## Acknowledgments

We are grateful to all authors that contributed to this Research Topic and to Samuel Oliver for editorial assistance.

## Conflict of interest

The authors declare that the research was conducted in the absence of any commercial or financial relationships that could be construed as a potential conflict of interest.

## Publisher’s note

All claims expressed in this article are solely those of the authors and do not necessarily represent those of their affiliated organizations, or those of the publisher, the editors and the reviewers. Any product that may be evaluated in this article, or claim that may be made by its manufacturer, is not guaranteed or endorsed by the publisher.
